# The Effect of Incentives and Meta-incentives on the Evolution of Cooperation

**DOI:** 10.1371/journal.pcbi.1004232

**Published:** 2015-05-14

**Authors:** Isamu Okada, Hitoshi Yamamoto, Fujio Toriumi, Tatsuya Sasaki

**Affiliations:** 1 Department of Business Administration, Soka University, Tokyo, Japan; 2 Department of Information Systems and Operations, Vienna University of Economics and Business, Vienna, Austria; 3 Department of Business Administration, Rissho University, Tokyo, Japan; 4 Department of Systems Innovations, University of Tokyo, Tokyo, Japan; 5 Faculty of Mathematics, University of Vienna, Vienna, Austria; University of Washington, UNITED STATES

## Abstract

Although positive incentives for cooperators and/or negative incentives for free-riders in social dilemmas play an important role in maintaining cooperation, there is still the outstanding issue of who should pay the cost of incentives. The second-order free-rider problem, in which players who do not provide the incentives dominate in a game, is a well-known academic challenge. In order to meet this challenge, we devise and analyze a meta-incentive game that integrates positive incentives (rewards) and negative incentives (punishments) with second-order incentives, which are incentives for other players’ incentives. The critical assumption of our model is that players who tend to provide incentives to other players for their cooperative or non-cooperative behavior also tend to provide incentives to their incentive behaviors. In this paper, we solve the replicator dynamics for a simple version of the game and analytically categorize the game types into four groups. We find that the second-order free-rider problem is completely resolved without any third-order or higher (meta) incentive under the assumption. To do so, a second-order costly incentive, which is given individually (peer-to-peer) after playing donation games, is needed. The paper concludes that (1) second-order incentives for first-order reward are necessary for cooperative regimes, (2) a system without first-order rewards cannot maintain a cooperative regime, (3) a system with first-order rewards and no incentives for rewards is the worst because it never reaches cooperation, and (4) a system with rewards for incentives is more likely to be a cooperative regime than a system with punishments for incentives when the cost-effect ratio of incentives is sufficiently large. This solution is general and strong in the sense that the game does not need any centralized institution or proactive system for incentives.

## Author Summary

Although social dilemmas can be resolved if punishing non-cooperators or rewarding cooperators works, such rewards and punishments, i.e., external incentives, entail certain expenses. As a result, a cooperative player who shirks his or her duty to provide an incentive to other players will emerge, and he or she will be more advantageous than an incentive-provider. In fact, the problem of excluding such cooperative incentive-non-providers, or second-order free-riders, is a well-known academic challenge. In order to meet this challenge, we devise and analyze a meta-incentive game that integrates positive incentives (rewards) and negative incentives (punishments) with second-order incentives, which are incentives for other players’ incentives. In this paper, we solve the replicator dynamics for a simple version of the game and analytically categorize the game types into four groups. We show that second-order incentives for first-order reward are necessary for cooperative regimes. This solution is general and strong in the sense that the game does not need any centralized institution or proactive system for incentives.

## Introduction

Even though society is based on cooperation, achieving cooperation in social dilemmas is still a big challenge. The free-rider problem, for example, hinders cooperation. Many studies have addressed this problem, and the methods proposed for solving it include giving players sufficient ability to remember their direct [1] or indirect experiences [2]. The idea is that this additional information generates cooperation through direct or indirect reciprocity. Other research has attempted to solve the problem by assigning tags [3], reputations [4], spatial structures [5] or networks [6] to players in the game. A third approach is to give players choices other than simply whether or not to contribute. A drawback of this approach, however, is that there may be a loner, i.e., a player who does not participate in the game [7, 8], or a joker, i.e., a destroyer who damages the public good [9]. Some researchers have devised another sort of game that promotes cooperation by giving players incentives explicitly. Important incentives for the evolution of cooperation are rewards and punishments as they tend to capture strong views of human nature [10, 11]. The approach we took integrates positive incentives such as rewarding and negative ones such as punishing into a system for promoting cooperation.

A meta-analysis of reviews on reward and punishment systems using a common framework [12] revealed that this approach is still controversial. Here, we tackle this issue by focusing on three perspectives: (1) the contrast between an individual incentive system and a centralized institutional one, (2) an incentive-integrated punishment and reward system, and (3) incentives on a meta-level.

First, many studies have focused on either individually-dealt-with punishments or rewards. While some studies [13–16] have shown that a costly punishment can effectively achieve cooperation, others [17–21] have shown just the opposite. Some researchers claim that the findings of peer-punishment studies may not be broadly applicable to modern human societies, because rewards and punishments are typically carried out by rules-bound institutions [22, 23] rather than by individuals. The reason might be a difficulty of establishing and maintaining such a peer-punishment system because it does not involve a nomocracy, i.e., a proactive or ex-ante commitment.

Second, should a free-rider be punished and/or should a contributor be rewarded? Experimental studies [24, 25] have indicated that rewards and punishments induce similar levels of cooperation when the incentive is very large. Economists have used experimental games to study the effects of positive and negative incentives (i.e., rewards and punishments) on the propensity to collaborate [26]. A theoretical study [27] showed that a punishment is more effective than a reward because these incentives have an asymmetric relationship with one another. In other words, punishments are not needed once cooperation is established, and thus, cooperators do not need to pay the costs of punishment. On the other hand, attaining rewards requires participants to pay costs by following a cooperative strategy. The threat of a strong punishment can achieve cooperation at a very low cost [28]. For an intermediate level of incentive, however, although punishments can induce greater cooperation than rewards [25], they cannot do so consistently [29]. Moreover, cooperation easily breaks down if both forms of incentive are removed [30]. Compared with the numerous studies on punishments, there have been relatively few on rewards [12, 24, 25, 29]. For example, an experimental study [31] explored the situation in which unkind newcomers are strictly exploited and found that indirect rewards are effective in such situations.

Third, in the approach we took, the focus is on meta-level incentives. When the incentive for cooperation is either a punishment or reward, there is still the second-order free-rider problem. The effort made to maintain a cooperative society is a cost that must be defrayed by someone. Generally, a player who contributes to a game but never defrays the cost for providing incentives is more evolutionarily adaptive than one who contributes to the game and does pay the cost. This means that eventually no one defrays the cost of maintaining the incentive system. The second-order free-rider problem can come down to the problem of costly incentives [32]. One solution is to implement a second-order incentive system.

The pioneering work on meta-level incentives was performed by Axelrod [33]. He attempted to evolve cooperation by imposing a second-order punishment on those who do not impose a first-order punishment when one is called for. His model linked punishments against non-punishers with punishments against non-cooperators. This assumption, that first-order incentives and second-order ones are linked, or FO-SO-linkages, is critical for our study. Yamagishi and Takahashi [34] were the first to point out the linkage issue and demonstrated that a cooperative regime emerges if it is assumed that players have linkages between cooperation and first-order incentives, or C-FO-linkages. Related studies have developed models that assume C-FO-linkages have been analyzed [35, 36]. The existence of a C-FO-linkage, however, is still not a foregone conclusion [37]. Some experimental studies [38, 39] showed that sanctions enhance norm and cooperative behavior. An experimental study [40], conversely, concluded there is a negative correlation between cooperative behaviors in prisoner’s dilemma games and refusal behaviors in ultimatum games. Moreover, an analysis of large-scale panel data of Germany [41] concluded that rewards and punishments have no relationship. Another experiment [42] found that cooperation is not correlated with norm-enforcing punishments. An experimental study [37], on the other hand, showed a significant correlation between cooperation and punishment; however, they unostentatiously admitted that their result was insufficient evidence for the existence of C-FO-linkages.

The C-FO-linkage issue is a relationship between two behaviors which differ qualitatively. Considering this point, the relationship between first-order incentives and second-order incentives is an alternative issue because they are both incentives. Kiyonari and Barclay [43] focused on the FO-SO-linkage and showed its existence in their experiments on one-shot public good games. Their study opened the door on analyses of models that assume FO-SO-linkages. Hilbe et al [44] experimentally showed the rationality of a second-order punishment in an authorized sanction system. We, in this paper, test a model that assumes a FO-SO-linkage in a peer-to-peer incentive system.

We have developed a model of a meta-incentive game (MIG) that has second-order incentives, i.e., incentives for other players to provide incentives. This analytical model can describe the carrot-and-stick issue uniformly and comparatively. The model targets an individual incentive system that integrates a positive side (reward) and a negative side (punishment) [45]. The incentive we consider is an ex-post type applied after players engage in donation games, and thus, no centralized institution with incentives is needed. An institution requires an ex-ante commitment among players. Players should decide whether or not they will participate in the institution before playing the donation games [23, 46]. In order to resolve the second-order free-rider problem, we suppose that there are three types of players in the MIG, i.e., a non-cooperative incentive-non-provider as a first-order free-rider, a cooperative incentive-non-provider as a second-order free-rider, and a cooperative incentive-provider, and we will explore the conditions under which cooperative incentive-providers survive.

## Results

MIG players first play donation games and then provide incentives in answer to their actions in the games. Incentives are provided not only for or against the others’ cooperative or non-cooperative actions but also for the others’ incentive behaviors or lack thereof on third-party players.

**Fig 1:**
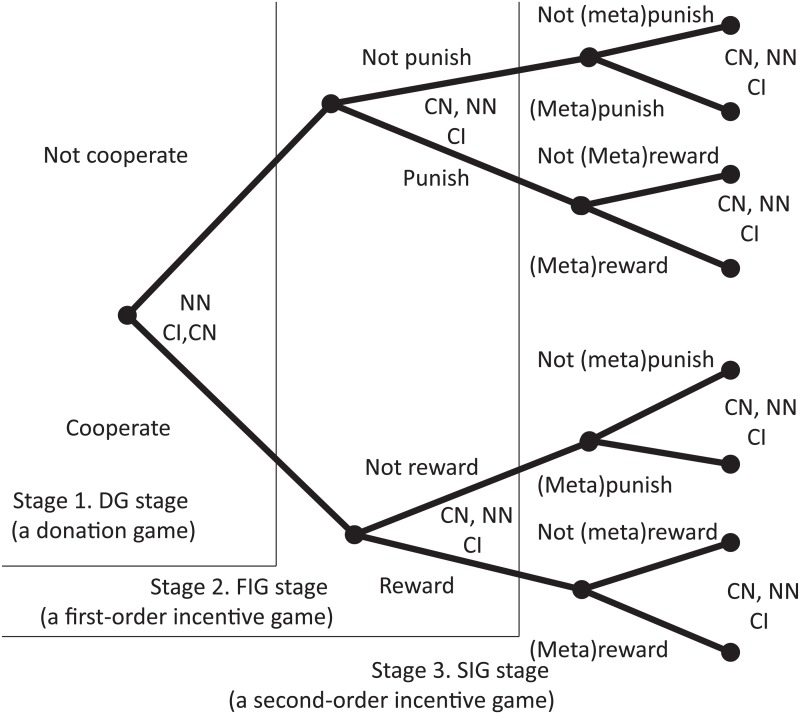
Illustration of meta-incentive game (MIG). Four individuals are randomly drawn from the population and randomly assigned to one of four roles, recipient, donor, first-order player, and second-order player. In the first stage, the donor decides whether to help the recipient. In the second stage, the first-order player decides whether to provide an incentive for the donor; and in the last stage, the second-order player decides whether to provide an incentive to the first-order player.

Players are divided into three sorts of strategist: a cooperative incentive-provider (CI), a cooperative incentive-non-provider (CN), and a non-cooperative incentive-non-provider (NN). Fig 1 shows an illustration of the MIG. The MIG consists of three stages (games): a donation game (DG), a first-order incentive game (FIG), and a second-order incentive game (SIG). Note that each player has perfect information, so each one knows all the players’ actions.

The population is infinitely large and well-mixed. The frequencies of the three strategies follow replicator dynamics describing a natural law whereby the higher one’s payoff is the more frequent one’s strategy becomes. Let *x*, *y*, and *z* be the frequencies of CI, CN, and NN, respectively. Naturally, *x*+*y*+*z* = 1. The equations are formulated as

$$\begin{array}{c} \dot{x}=x\left(U_{CI}-\bar{U}\right),\\ \dot{y}=y\left(U_{CN}-\bar{U}\right),\\ \dot{z}=z\left(U_{NN}-\bar{U}\right), \end{array}$$ (1)

where *U*_*CI*_, *U*_*CN*_, *U*_*NN*_, and $\bar{U}$ are, respectively, the average payoffs of CI, CN, NN, and all the players. $\bar{U}$ is given by

$$\begin{array}{c} \bar{U}=xU_{CI}+yU_{CN}+zU_{NN}. \end{array}$$ (2)

Now let us describe the parameter notations needed to calculate a player’s (expected) payoff. Let *c* be the cost of donation, *b* be the receiver’s benefit, *F*_1_ be the fine imposed as a first-order punishment, *P*_1_ be the cost of a first-order punishment, *A*_1_ be the amount of a first-order reward, *R*_1_ be the cost of a first-order reward, *F*_*P*_ be the fine for freeriding for the first-order punishment, *P*_*P*_ be the cost for freeriding for the first-order punishment, *A*_*P*_ be the amount of the reward for the punisher, *R*_*P*_ be the cost of the reward for the punisher, *F*_*R*_ be the fine for freeriding for giving a reward, *P*_*R*_ be the cost for freeriding for giving a reward, and *A*_*R*_ be the amount of rewarding a rewarder, and *R*_*R*_ be the cost of rewarding a rewarder. All these values should be non-negative constants.

We will avoid analytical difficulties due to the usage of many parameters by defining a simple meta-incentive game (S-MIG) using two parameters: the incentive cost-effect ratio (*μ*), which represents the proportion of a fine or award that incentive-receivers should pay or receive relative to its cost, and the discount factor of costs on the level of incentive (*δ*), where

$$\begin{array}{c} \mu =\frac{F_{1}}{P_{1}}=\frac{F_{P}}{P_{P}}=\frac{F_{R}}{P_{R}}=\frac{A_{1}}{R_{1}}=\frac{A_{R}}{R_{R}}=\frac{A_{P}}{R_{P}}, \end{array}$$

and

$$\begin{array}{c} \delta =\frac{P_{1}}{c}=\frac{R_{1}}{c}=\frac{P_{P}}{P_{1}}=\frac{R_{R}}{R_{1}}=\frac{P_{R}}{P_{1}}=\frac{R_{P}}{R_{1}}. \end{array}$$

We assume that *μ* > 1 and 0 < *δ* < 1. We can set *c* = 1 without loss of generality. An S-MIG is perfectly described by a duplet (*μ*, *δ*).

**Fig 2:**
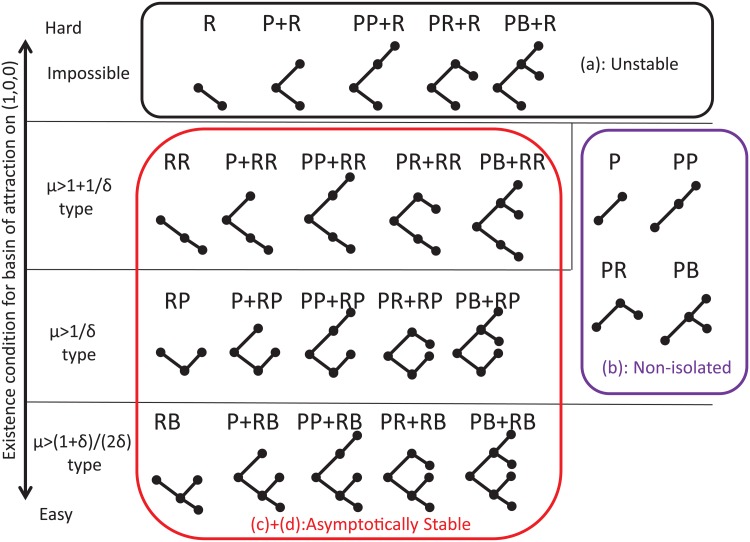
Illustration of replicator dynamics analyses for each type of S-MIG. This figure illustrates all 24 types of S-MIG. The abbreviations are defined in Table 1. Their vertical layering in the figure reflects the existence condition for the basin of attraction on the point (*x*, *y*, *z*) = (1, 0, 0) related to (*μ*, *δ*) under which a cooperative regime emerges. The frames represent the form of local stability at point (*x*, *y*, *z*) = (1, 0, 0): the point is unstable for each type in the top frame which corresponds to (A) in Fig 3, is a non-isolated equilibrium for each type in the bottom right frame which corresponds to (B) in Fig 3, and is asymptotically stable for each type in the bottom left frame which corresponds to (C) and (D) in Fig 3.

Finally, before analyzing our model, we define all 24 possible configurations of MIG in Fig 2. For example, the P-type MIG has only first-level punishments. In this type, players can give or receive neither a first-level reward nor a second-level incentive. The PR type has first-level punishments for non-cooperators and second-level rewards for punishers.

**Fig 3:**
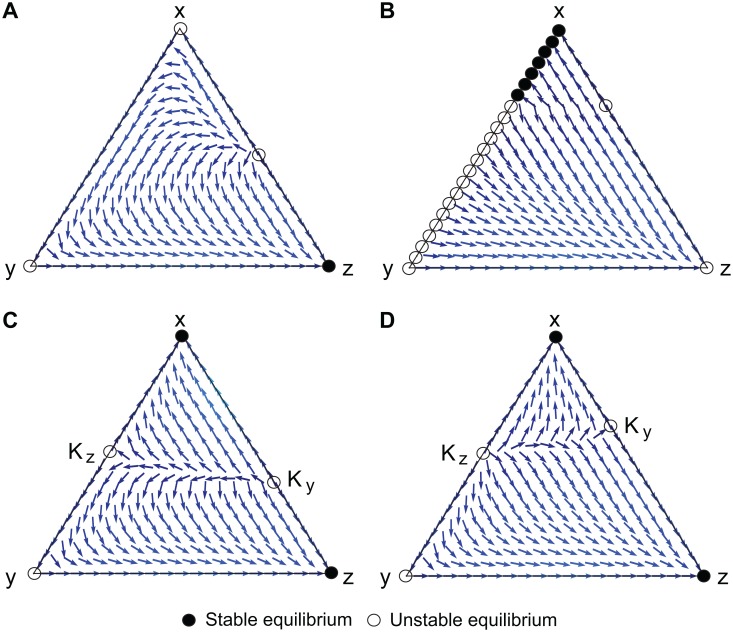
Replicator dynamics analysis of representative S-MIGs on 2-dimensional simplex. The triangle represents the state space, Δ = {(*x*, *y*, *z*)*** : *x*, *y*, *z* ≥ 0, *x*+*y*+*z* = 1}, where *x*, *y*, and *z* are respectively the frequencies of the cooperative incentive-providers, cooperative incentive-non-providers, and non-cooperative incentive-non-providers. $\left(\mu ,\delta )=(3,\frac{1}{2}\right)$. (A) PR+R, (B) PP, (C) PB+RB(Full), and (D) RB. The abbreviations are defined in Table 1. In (A), (*x*, *y*, *z*) = (1, 0, 0) is unstable, so cooperation is never achieved regardless of the values of (*μ*, *δ*). In (B), the whole line *z* = 0 consists of fixed points, and thus, neutral drift is possible. In (C) and (D), (*x*, *y*, *z*) = (1, 0, 0) is a locally asymptotically stable point depending on the values of (*μ*, *δ*), and thus, a cooperative regime can emerge. In (C), the unstable equilibrium in the internal part on *z* = 0, *K*_*z*_, is a saddle, and that on *y* = 0, *K*_*y*_, is a source. In (D), *K*_*z*_ is a source, while *K*_*y*_ is a saddle.

We explore the conditions under which a cooperative equilibrium (*x* > 0) emerges by analyzing the replicator dynamics on different types of S-MIG. As shown in Methods, the dynamics of S-MIGs can be classified into four groups. Fig 2 illustrates the existence condition for the basin of attraction and the local stabilities on the point (*x*, *y*, *z*) = (1, 0, 0) of all types, and Fig 3 shows the phase portraits of the representative S-MIGs on a 2-dimensional simplex.

We identified certain features of the second-order free-rider problem. First, as the second-order free-rider problem warns, cooperation cannot be maintained in any system with only first-order incentives. Similarly, cooperation does not arise even if the system has second-order incentives but no second-order incentives for first-order rewards. This is because rewarding others is equivalent to a prisoner’s dilemma game or a donation game, so incentive-providers as well as cooperators diminish over time.

Second, because of the neutral drift effect, no system without a reward side can keep its position when cooperation dominates. This is another aspect of the second-order free-rider problem. These results reveal two important facts about reward systems: a second-order incentive system for first-order rewards is better than a non-incentive system on the reward side for promoting cooperation, and a system without second-order incentives for first-order rewards is worse than the non-incentive system.

Third, any system with second-order incentives for first-order rewards can produce a stable cooperative regime under specific conditions. To do so, a certain number of cooperative incentive-providers are needed. If their numbers are small, they cannot survive.

Fourth, the conditions under which a cooperative regime emerges depend on the system. A system with second-order rewards has the strictest condition, and the conditions become less stringent for a system with second-order punishments and a system with second-order both rewards and punishments.

Finally, the condition on the frequency of incentive-providers under which a cooperative regime can be sustained also depends on the system. When the cost-effect ratio of incentives is sufficiently large, the lower limit of the frequency of incentive-providers in a system with second-order rewards for first-order incentives is lower than that in a system with second-order punishments for first-order incentives.

## Discussion

What can resolve the second-order free-rider problem? Sigmund et al [23] achieved stabilizing cooperative regimes by using pool punishments instead of peer punishments. Is it possible to maintain such regimes without any proactive institution? Our model demonstrates that assuming first-order and second-order incentives are linked can lead to a solution without any social costs or a punishment fund. The assumed linkage means that individuals who are willing to provide incentives would automatically provide meta-incentives as well. The consequences are that although the model allows second-order free riders, it does not allow third-order free riders. (If they were allowed, they would again destabilize cooperation.) Moreover, efficiency is traded for stability in Sigmund et al’s model, because individuals pay for a punishment fund without as yet knowing who the free riders to be punished are [23]. In cotrast, our model can search for an efficient incentive level for maintaining cooperative regimes, because individuals can reactively incur costs for incentives with knowing who is to be given an incentive. Thus, one of the implications of the linkage assumption is that a more efficient incentive system for stabilizing cooperation would be an intermediate of the traditional peer and pool incentive systems.

We note that a similar amount of cooperation could also be achieved if there was a linkage between cooperation and first-order incentives (C-FO linkage, see Methods). This linkage means an alternative model with two strategies, i.e., defectors and cooperators who automatically also provide first-order incentives. As shown in Methods, our model can indeed cover a case that assumes C-FO linkages instead of FO-SO linkages in specific parameters.

For our model incentives for reward are necessary for cooperative regimes. If players play in a non-incentive system on the reward side, a punishment function does not work when cooperation is achieved. That is to say, they cannot respond to an invasion of neutral mutants who do not provide incentives. As a result, cooperation suddenly collapses. This is why Axelrod’s simulation [33] cannot keep a cooperative regime for a long time [47–49]. Therefore, if players play in a non-incentive system on the reward side, another mechanism is needed, e.g., a social vaccine proposed by Yamamoto and Okada [50], to maintain cooperative regimes.

How about a system with first-order reward and without second-order incentives for the reward? Here, worse comes to worst because it becomes free from the possibility of staying a cooperative regime temporally. When a cooperative regime is achieved, players do not need their punishment functions, and thus, only the first-order reward function works. As the second-order free-rider problem indicates, cooperative incentive-non-providers beat cooperative incentive-providers because they don’t bear the burden of paying for rewards.

Second-order incentives for first-order rewards are necessary for achieving and maintaining robust cooperative regimes and to resolve the second-order free-rider problem. That is, a mechanism is needed to make it beneficial for a player to give a first-order reward. Assuming that players who tend to provide incentives for other players’ cooperative or non-cooperative behaviors also tend to provide incentives for their incentive behaviors, the second-order free-rider problem can be completely resolved without any third-order or higher (meta)incentive. In our model, moreover, incentives are performed ex-post and individually, and thus the system does not need a centralized institution or ex-ante commitment. Many studies on non-meta-level incentives have shown that punishment is more effective than a reward. We have identified a possible explanation as to why people prefer second-order rewards to second-order punishments. Kiyonari and Barclay’s experimental study [43] supports this—they found that people readily provided second-order rewards toward those who rewarded cooperators while they did not administer second-order punishments to non-punishers because the reward systems were more easily supported by higher order incentives and were thus more likely to persist.

Which incentive should a designer of an MIG choose? If the cost-effect ratio of incentives is sufficiently large, even a handful of cooperative incentive-providers can beat non-cooperative incentive-non-providers if the designer uses a system with rewards for incentives instead of a system with punishments for incentives.

Our work differs from Sasaki et al’s model of integrating rewards and punishments, which was designed for an institutional system with a compulsory entrance fee and thus no option of second-order free riders [51]. Kendal et al [52] analyzed a model of second-order peer rewards for punishers, but did not consider rewards for cooperators who contribute in the game. Our paper is a pioneering analysis of MIGs; as such, we only dealt with a minimum deviation and analyze pro-social incentives and left anti-social punishments [53] and anti-social rewards as topics for future study. Our model assumes an infinitely well-mixed population, and this assumption should be loosened in the future. Szolnoki and Perc [54] compared the effect of a reward with that of a punishment in a spatial public goods game. Chen and Perc [55] studied the optimal distribution of institutional incentives in a public goods game on a scale-free network.

The asymmetry of the effects of rewards and punishments might be evident in the cost-effect ratio. Although it may be natural that a fine handed out as punishment should be larger than its cost, can the reward be larger than its cost? We believe that this balances out the risk of second-order free-riders emerging. Of course, we leave open the possibility of incorporating asymmetric reward and punishment effects as a future extension of a system.

## Methods

In this section, we analyze a meta-incentive game (MIG) by solving replicator equations. Table 1 gives brief descriptions of all 24 types of the game.

**Table 1:** Types of MIG.

Type	Brief description	Parameters used
P	Punishment for non-cooperators on 1st level	*F*_1_, *P*_1_
R	Reward for cooperators on 1st level	*A*_1_, *R*_1_
P+R	Both reward and punishment on 1st level (as well as P-type plus R-type)	*F*_1_, *P*_1_, *A*_1_, *R*_1_
PP	Punishment for non-cooperators on 1st level and punishment for non-punishers on 2nd level	*F*_1_, *P*_1_, *F*_*P*_, *P*_*P*_
PR	Punishment for non-cooperators on 1st level and reward for punishers on 2nd level	*F*_1_, *P*_1_, *A*_*P*_, *R*_*P*_
PB	Punishments on both levels (as well as PP-type) and reward for punishers on 2nd level	*F*_1_, *P*_1_, *F*_*P*_, *P*_*P*_, *A*_*P*_, *R*_*P*_
RP	Reward for cooperators on 1st level and punishment for non-rewarders on 2nd level	*A*_1_, *R*_1_, *F*_*R*_, *P*_*R*_
RR	Reward for cooperators on 1st level and reward for rewarders on 2nd level	*A*_1_, *R*_1_, *A*_*R*_, *R*_*R*_
RB	Rewards on both levels (as well as RR-type) and punishment for non-rewarders on 2nd level	*A*_1_, *R*_1_, *F*_*R*_, *P*_*R*_, *A*_*R*_, *R*_*R*_
P+RP	Both reward and punishment on 1st level and punishment for non-rewarders on 2nd level	*A*_1_, *R*_1_, *F*_1_, *P*_1_, *F*_*R*_, *P*_*R*_
P+RR	Both reward and punishment on 1st level and reward for rewarders on 2nd level	*A*_1_, *R*_1_, *F*_1_, *P*_1_, *A*_*R*_, *R*_*R*_
P+RB	Both reward and punishment on 1st level, and both punishment for non-rewarders and reward for rewarders on 2nd level	*A*_1_, *R*_1_, *F*_1_, *P*_1_, *A*_*R*_, *R*_*R*_, *F*_*R*_, *P*_*R*_
PP+R	Both reward and punishment on 1st level and punishment for non-punisher on 2nd level	*F*_1_, *P*_1_, *A*_1_, *R*_1_, *F*_*P*_, *P*_*P*_
PR+R	Both reward and punishment on 1st level and reward for punisher on 2nd level	*F*_1_, *P*_1_, *A*_1_, *R*_1_, *A*_*P*_, *R*_*P*_
PB+R	Both reward and punishment on 1st level, and both punishment for non-punishers and reward for punishers on 2nd level	*F*_1_, *P*_1_, *A*_1_, *R*_1_, *F*_*P*_, *P*_*P*_, *A*_*P*_, *R*_*P*_
PP+RP	Both reward and punishment on 1st level, and punishments for both non-punishers and non-rewarders on 2nd level	*F*_1_, *P*_1_, *A*_1_, *R*_1_, *F*_*P*_, *P*_*P*_, *F*_*R*_, *P*_*R*_
PP+RR	Both reward and punishment on 1st level, and both punishment for non-punishers and reward for rewarders on 2nd level	*F*_1_, *P*_1_, *A*_1_, *R*_1_, *F*_*P*_, *P*_*P*_, *A*_*R*_, *R*_*R*_
PP+RB	Full-type excluded reward for punishers on 2nd level	All except *A*_*P*_, *R*_*P*_
PR+RP	Both reward and punishment on 1st level, and both punishment for non-rewarders and reward for punishers on 2nd level	*F*_1_, *P*_1_, *A*_1_, *R*_1_, *F*_*R*_, *P*_*R*_, *A*_*P*_, *R*_*P*_
PR+RR	Both reward and punishment on the 1st level, and rewards for both punishers and rewarders on the 2nd level	*A*_1_, *R*_1_, *F*_1_, *P*_1_, *A*_*R*_, *R*_*R*_, *A*_*P*_, *R*_*P*_
PR+RB	The Full-type excluded punishment for non-punishers on 2nd level	All except *F*_*P*_, *P*_*P*_
PB+RP	Full-type excluded reward for rewarders on 2nd level	All except *A*_*R*_, *R*_*R*_
PB+RR	Full-type excluded punishment for non-rewarders on 2nd level	All except *F*_*R*_, *P*_*R*_
PB+RB (Full)	MIG itself	All

### Expected payoffs of the players

The expected payoffs of the players are

$$\begin{array}{ccc} U_{CI} & = & b\left(x+y\right)-c-\left[\left(x+y\right)R_{1}+zP_{1}\right]+xA_{1}\\ & & -\;[\left(x+y\right)xR_{R}+\left(x+y\right)\left(y+z\right)P_{R}+zxR_{P}\\ & & +\;z\left(y+z\right)P_{P}]+x\left[\left(x+y\right)A_{R}+zA_{P}\right],\\ U_{CN} & = & b\left(x+y\right)-c+xA_{1}-x\left[\left(x+y\right)F_{R}+zF_{P}\right],\\ U_{NN} & = & b\left(x+y\right)-xF_{1}-x\left[\left(x+y\right)F_{R}+zF_{P}\right]. \end{array}$$ (3)

Let us explain the terms of *U*_*CI*_. The first term represents the benefit of donation, whereas the second term gives the cost of donation (because one is a cooperator) in the DG stage. The third term represents the costs of the first-order incentivization: rewarding a cooperator and punishing a non-cooperator. The fourth term represents the first-order reward for cooperating. The fifth term represents the costs of the second-order incentivization and consists of four parts, i.e., rewarding those who have rewarded a cooperator, punishing those who have not rewarded a cooperator, rewarding those who have punished a non-cooperator, and punishing those who have not punished a non-cooperator. Finally, the last term describes the second-order rewards for rewarding a cooperator and punishing a non-cooperator. Similar explanations can be applied to the other expected payoffs, *U*_*CN*_ and *U*_*NN*_.

### Replicator dynamics analysis for various types of S-MIG

Using Eqs (1), (2), and (3), the replicator equations are calculated as $\dot{x}=xf_{1},\dot{y}=yf_{2}$, and $\dot{z}=zf_{3}$, where

$$\begin{array}{ccc} f_{1} & = & -cz-\left(y+z\right)\left[\left(x+y\right)R_{1}+zP_{1}\right]+xz\left(A_{1}+F_{1}\right)\\ & & -\;\left(y+z\right)[\left(x+y\right)xR_{R}+\left(x+y\right)\left(y+z\right)P_{R}+zxR_{P}\\ & & +\;z\left(y+z\right)P_{P}]+x[\left(x+y\right)\left(y+z\right)\left(A_{R}+F_{R}\right)\\ & & +\;z\left(y+z\right)\left(A_{P}+F_{P}\right)],\\ f_{2} & = & -cz+x\left[\left(x+y\right)R_{1}+zP_{1}\right]+xz\left(A_{1}+F_{1}\right)\\ & & +\;x[\left(x+y\right)xR_{R}+\left(x+y\right)\left(y+z\right)P_{R}+zxR_{P}\\ & & +\;z\left(y+z\right)P_{P}]-x[\left(x+y\right)x\left(A_{R}+F_{R}\right)\\ & & +\;zx(A_{P}+F_{P})],\\ f_{3} & = & c\left(x+y\right)+x\left[\left(x+y\right)R_{1}+zP_{1}\right]-x\left(x+y\right)\left(A_{1}+F_{1}\right)\\ & & +\;x[\left(x+y\right)xR_{R}+\left(x+y\right)\left(y+z\right)P_{R}+zxR_{P}\\ & & +\;z\left(y+z\right)P_{P}]-x[\left(x+y\right)x\left(A_{R}+F_{R}\right)\\ & & +\;zx(A_{P}+F_{P})]. \end{array}$$ (4)

We prove that there is no equilibrium at any internal point (*x*, *y*, *z*) in S-MIG in the following subsection, and thus, here, it is enough to conduct an analysis of the borders. Moreover, there is no equilibrium at any point on the line *x* = 0, except two corners ((*y*, *z*) = (0, 1), (1, 0)). This is because, $\dot{y}=-y(1-y)<0$ on the line *x* = 0, and thus, *y* always decreases. Hence, we will only deal with the $\dot{x}$ function on the lines *y* = 0 and *z* = 0. On those two lines, that function is calculated as ${\dot{x}|}_{y=0}=x(1-x)f(x)$ and ${\dot{x}|}_{z=0}=x(1-x)g(x)$, where

$$\begin{array}{ccc} f(x) & = & -1-xR_{1}-\left(1-x\right)P_{1}+x\left(A_{1}+F_{1}\right)-x^{2}R_{R}\\ & & -\;x\left(1-x\right)P_{R}-x\left(1-x\right)R_{P}-{\left(1-x\right)}^{2}P_{P}\\ & & +\;x^{2}\left(A_{R}+F_{R}\right)+x\left(1-x\right)\left(A_{P}+F_{P}\right),\\ g(x) & = & -R_{1}-xR_{R}-\left(1-x\right)P_{R}+xA_{R}+xF_{R}. \end{array}$$ (5)

In order to exemplify how to analyze each type of the S-MIG, we deal with the RB type as a representative and derive their equations of *f*(*x*) and *g*(*x*). In the type, *F*_1_, *P*_1_, *F*_*P*_, *P*_*P*_, *A*_*P*_ and *R*_*P*_ should be zero because there has no incentive system on the punishment side. Moreover, using *c* = 1 and definitions of *μ* and *δ*, *R*_1_ = *δ*, *A*_1_ = *μδ*, *R*_*R*_ = *P*_*R*_ = *δ* ^2^ and *A*_*R*_ = *F*_*R*_ = *μδ* ^2^. Substituting them into *f*_1_ in Eq (4), we get

$$\begin{array}{c} \dot{x}=x\left[-z-\delta \left(y+z\right)\left(x+y\right)+\mu \delta xz-\delta^{2}\left(y+z\right)\left(x+y\right)+2\mu \delta^{2}x\left(x+y\right)\left(y+z\right)\right]. \end{array}$$

Therefore, we derive Eq (5) of the RB-type S-MIG as

$$\begin{array}{c} {\dot{x}|}_{y=0}=x\left(1-x\right)\left(-1-\delta x+\mu \delta x-\delta^{2}x+2\mu \delta^{2}x^{2}\right), \end{array}$$

and

$$\begin{array}{c} {\dot{x}|}_{z=0}=x\left(1-x\right)\left(-\delta -\delta^{2}+2\mu \delta^{2}x\right). \end{array}$$

Table 2 shows the equations for ${\dot{x}|}_{y=0}$ and ${\dot{x}|}_{z=0}$ for each type of S-MIG. Using Eq (5), we can calculate an existence condition for the basin of attraction on the point (*x*, *y*, *z*) = (1, 0, 0). If ${\dot{x}|}_{x=1,y=0}>0$ and ${\dot{x}|}_{x=1,z=0}>0$ are satisfied, the point (*x*, *y*, *z*) = (1, 0, 0) is asymptotically stable, and thus, a cooperative regime emerges. The dynamics of each type of S-MIG are classified into four groups. In what follows, we will deal with one representative type in each group. The remainder can be similarly derived.

**Table 2:** $\dot{x}$ functions on the lines *y* = 0 and *z* = 0 for each type of S-MIG. Here, *f*(*x*) and *g*(*x*) are defined in Eq (5).

Type	*f*(*x*)	*g*(*x*)
P	−1−*δ*(1−*x*)+*μδx*	0
R	−1−*δx*+*μδx*	−*δ*
P+R	−1−*δ*+2*μδx*	−*δ*
PP	−1−*δ*(1−*x*)+*μδx*−*δ* ^2^(1−*x*)^2^+*μδ* ^2^ *x*(1−*x*)	0
PR	−1−*δ*(1−*x*)+*μδx*−*δ* ^2^ *x*(1−*x*)+*μδ* ^2^ *x*(1−*x*)	0
PB	−1−*δ*(1−*x*)+*μδx*−*δ* ^2^(1−*x*)+2*μδ* ^2^ *x*(1−*x*)	0
RP	−1−*δx*+*μδx*−*δ* ^2^ *x*(1−*x*)+*μδ* ^2^ *x* ^2^	−*δ*−*δ* ^2^(1−*x*)+*μδ* ^2^ *x*
RR	−1−*δx*+*μδx*−*δ* ^2^ *x* ^2^+*μδ* ^2^ *x* ^2^	−*δ*−*δ* ^2^ *x*+*μδ* ^2^ *x*
RB	−1−*δx*+*μδx*−*δ* ^2^ *x*+2*μδ* ^2^ *x* ^2^	−*δ*−*δ* ^2^+2*μδ* ^2^ *x*
P+RP	−1−*δ*+2*μδx*−*δ* ^2^ *x*(1−*x*)+*μδ* ^2^ *x* ^2^	−*δ*−*δ* ^2^(1−*x*)+*μδ* ^2^ *x*
P+RR	−1−*δ*+2*μδx*−*δ* ^2^ *x* ^2^+*μδ* ^2^ *x* ^2^	−*δ*−*δ* ^2^ *x*+*μδ* ^2^ *x*
P+RB	−1−*δ*+2*μδx*−*δ* ^2^ *x*+2*μδ* ^2^ *x* ^2^	−*δ*−*δ* ^2^+2*μδ* ^2^ *x*
PP+R	−1−*δ*+2*μδx*−*δ* ^2^(1−*x*)^2^+*μδ* ^2^ *x*(1−*x*)	−*δ*
PR+R	−1−*δ*+2*μδx*−*δ* ^2^ *x*(1−*x*)+*μδ* ^2^ *x*(1−*x*)	−*δ*
PB+R	−1−*δ*+2*μδx*−*δ* ^2^(1−*x*)+2*μδ* ^2^ *x*(1−*x*)	−*δ*
PP+RP	−1−*δ*+2*μδx*−*δ* ^2^(1−*x*)+*μδ* ^2^ *x*	−*δ*−*δ* ^2^(1−*x*)+*μδ* ^2^ *x*
PP+RR	−1−*δ*+2*μδx*−*δ* ^2^[*x* ^2^+(1−*x*)^2^]+*μδ* ^2^ *x*	−*δ*−*δ* ^2^ *x*+*μδ* ^2^ *x*
PP+RB	−1−*δ*+2*μδx*−*δ* ^2^[1−*x*(1−*x*)]+*μδ* ^2^ *x*(*x*+1)	−*δ*−*δ* ^2^+2*μδ* ^2^ *x*
PR+RP	−1−*δ*+2*μδx*−2*δ* ^2^ *x*(1−*x*)+*μδ* ^2^ *x*	−*δ*−*δ* ^2^(1−*x*)+*μδ* ^2^ *x*
PR+RR	−1−*δ*+2*μδx*−*δ* ^2^ *x*+*μδ* ^2^ *x*	−*δ*−*δ* ^2^ *x*+*μδ* ^2^ *x*
PR+RB	−1−*δ*+2*μδx*−*δ* ^2^ *x*(2−*x*)+*μδ* ^2^ *x*(*x*+1)	−*δ*−*δ* ^2^+2*μδ* ^2^ *x*
PB+RP	−1−*δ*+2*μδx*−*δ* ^2^(1−*x*)(*x*+1)+*μδ* ^2^ *x*(2−*x*)	−*δ*−*δ* ^2^(1−*x*)+*μδ* ^2^ *x*
PB+RR	−1−*δ*+2*μδx*−*δ* ^2^[1−*x*(1−*x*)]+*μδ* ^2^ *x*(2−*x*)	−*δ*−*δ* ^2^ *x*+*μδ* ^2^ *x*
PB+RB	−1−*δ*+2*μδx*−*δ* ^2^+2*μδ* ^2^ *x*	−*δ*−*δ* ^2^+2*μδ* ^2^ *x*

The R, P+R, PP+R, PR+R, and PB+R types belong in the first group. Each type of this group has a globally stable point (*x*, *y*, *z*) = (0, 0, 1), so cooperation is never achieved regardless of the values of (*μ*, *δ*). ${\dot{x}|}_{z=0}<0$ is satisfied; thus, (1, 0, 0) is unstable. Therefore, the only stable equilibrium point is (0, 0, 1), as shown in Fig 3(A).

The P, PP, PR, and PB types belong in the second group. The whole line *z* = 0 consists of fixed points because ${\dot{x}|}_{z=0}=0$ is always satisfied. Each type of this group has two patterns of behavior depending on the values of (*μ*, *δ*). In one, all the fixed points on the line *z* = 0 are unstable, and thus, there is a globally stable point (0, 0, 1), as in the first group. In the other, some of the fixed points are stable if $x>\frac{1}{\mu \delta }$ when $\mu >\frac{1}{\delta }$, as shown in the following subsection. Note that this behavior also satisfies an existence condition for the basin of attraction on the point (*x*, *y*, *z*) = (1, 0, 0) on *y* = 0. Let us verify the PR case as a representative example. $\frac{\partial f^{2}}{\partial^{2}x}(x)=2\delta^{2}(1-\mu )<0$ and $\frac{\partial f}{\partial x}(1)=\mu \delta (1-\delta )+\delta (1+\delta )>0$ prove that *f*(*x*) is an increasing function. Moreover, *f*(1) = −1+*μδ* > 0 implies that the point (*x*, *y*, *z*) = (1, 0, 0) is asymptotically stable. Even in the second pattern, however, the point (1, 0, 0) is not stable for a long time. This is because, the whole line *z* = 0 consists of fixed points, and thus, neutral drift is possible. Occasionally, *x* moves to an unstable equilibrium, and this type eventually reaches (0, 0, 1). The phase diagram of the PP-type S-MIG is shown in Fig 3(B).

The third group, consisting of types which include either the RP or RR, and P+RB, PP+RB, PR+RB, and PB+RB (Full) types, has two patterns of behavior depending on the values of (*μ*, *δ*). In one, there is a globally stable point (0, 0, 1), as in the first group. In the other, another locally asymptotically stable point (1, 0, 0) can exist. This group has three existence conditions for the basin of attraction on the point (*x*, *y*, *z*) = (1, 0, 0): $\mu >1+\frac{1}{\delta }$ for the types which include RR, $\mu >\frac{1}{\delta }$ for the types which include RP, and $\mu >\frac{1+\delta }{2\delta }$ for the P+RB, PP+RB, PR+RB, and PB+RB (Full) types. Let us examine the PB+RB(Full)-type S-MIG as a representative type. Here, ${\dot{x}|}_{z=0}=\delta x(1-x)(2\mu \delta x-\delta -1)$; hence, the dynamics on the line *z* = 0, on one hand, are bistable and $x=\frac{1+\delta }{2\mu \delta }$ is a fixed point that separates the two basins of attraction when $\mu >\frac{1+\delta }{2\delta }$ is satisfied. On the other hand, the dynamics on *y* = 0 depends on *f*(*x*). $\frac{\partial f}{\partial x}(x)=2\mu \delta (1+\delta )>0$ shows that ${\dot{x}|}_{y=0}$ is an increasing function. *f*(0) < 0 and *f*(1) = −1−*δ*−*δ* ^2^+2*μδ*(1+*δ*) > 0 when the existence condition for the basin of attraction on the point (*x*, *y*, *z*) = (1, 0, 0) is satisfied. Therefore, the dynamics on *y* = 0 are also bistable, and $x=\frac{1+\delta +\delta^{2}}{2\mu \delta (1+\delta )}$ is a fixed point that separates the two basins of attraction. Fig 3(C) shows the phase diagram of the Full-type S-MIG.

The final group, consisting of only the RB type, is the same as the third except for the direction of the dynamics in the internal space of the basin of attraction for cooperation (see Fig 3(D)). In this type, the unstable equilibrium point on the line *z* = 0 is a source while those in the third group are saddles. Likewise, the unstable equlibrium point on *y* = 0 in the RB type is a saddle, while those in the third group are sources. Using the analytical method of the following subsections, we can calculate the eigenvalues of the matrices derived by linearization of the dynamics around the equilibrium point. The eigenvalues of the equilibrium on the line *z* = 0 are $\frac{1-\delta }{2}$ and $\left(1-\frac{1+\delta }{2\mu \delta })\delta (1+\delta \right)$, and both are positive. Local stability theory says that an equilibrium with two positive eigenvalues is unstable and an equilibrium with one positive eigenvalue and one negative eigenvalue is a saddle. We can verify that the types of the third group have inverse stabilities. First, we deal with the Full-type S-MIG. Let *λ*_1_ and *λ*_2_ be the eigenvalues of the matrix derived by linearization of the dynamics around the equilibrium point (x*, y*, 0) on the line *z* = 0. *λ*_1_ *λ*_2_ = −y* *δ* ^2^(1+*δ*) < 0. Moreover, in the case of the RR-type S-MIG, *λ*_1_ *λ*_2_ = −x* y* *δ* ^2^ < 0. The other cases are omitted.

Finally, we compare the lower limits of *x* of the basins of attraction for cooperation (*x*, *y*, *z*) = (1, 0, 0) on *y* = 0. Let *f*_*D*_(*x*) and *x*_*D*_ be $\frac{{\dot{x}|}_{y=0}}{xz}$ and the lower limit of *x* of the basin of attraction on the line *y* = 0 in the *D*-type S-MIG for *D* ∈ {P, R, …, PB+RB}. Basically, the more complex the type is, the lower its lower limit of *x* becomes. For example, we will prove *x*_P+RB_ < *x*_P+RR_. *f*_P+RB_(*x*) = −1−*δ*+2*μδx*−*δ* ^2^ *x*+2*μδ* ^2^ *x* ^2^ and *f*_P+RR_(*x*) = −1−*δ*+2*μδx*−*δ* ^2^ *x* ^2^+*μδ* ^2^ *x* ^2^ are in Table 2. $\frac{\partial f_{\text{P+RB}}}{\partial x}(x)>0$ and $\frac{\partial f_{\text{P+RR}}}{\partial x}(x)>0$ imply that both functions are increasing. Note that *f*_*D*_(*x*_*D*_) = 0. *f*_P+RB_(*x*_P+RR_) = *δ* ^2^ *x*_P+RR_[*x*_P+RR_(1+*μ*)−1] and $f_{\text{P+RR}}(\frac{1}{1+\mu })<0$ then *f*_P+RB_(*x*_P+RR_) > 0. Therefore, *x*_P+RB_ < *x*_P+RR_. Moreover, some equivalence relations can be derived. For example, let us compare *x*_RR_ with *x*_RP_. *f*_RR_(*x*) and *f*_RP_(*x*) are increasing functions because their partial derivatives are positive in 0 < *x* < 1. *f*_RR_(*x*_RP_) = *δ* ^2^ *x*_RP_(1−2*x*_RP_) and $f_{\text{RP}}(\frac{1}{2})=\frac{\left(\mu -1)\delta (2+\delta \right)}{4}-1$ are derived. Therefore, if $\mu >\frac{4}{\delta (\delta +2)}+1$, then $x_{\text{RP}}<\frac{1}{2}$ and *x*_RR_ < *x*_RP_.

$$\begin{array}{ccc} \mu >\mu_{0} & \Leftrightarrow & x_{R}<x_{P},\\ \mu >\mu_{10} & \Leftrightarrow & x_{\mathrm{PR}}<x_{\mathrm{PP}}\Leftrightarrow x_{\mathrm{RR}}<x_{\mathrm{RP}},\\ \mu >\mu_{11} & \Leftrightarrow & x_{P+\mathrm{RR}}<x_{P+\mathrm{RP}}\Leftrightarrow x_{\mathrm{PR}+R}<x_{\mathrm{PP}+R},\\ \mu >\mu_{12} & \Leftrightarrow & x_{\mathrm{PP}+\mathrm{RR}}<x_{\mathrm{PP}+\mathrm{RP}}\Leftrightarrow x_{\mathrm{PR}+\mathrm{RR}}<x_{\mathrm{PR}+\mathrm{RR}}\\ & \Leftrightarrow & x_{\mathrm{PR}+\mathrm{RR}}<x_{\mathrm{PP}+\mathrm{RR}}\Leftrightarrow x_{\mathrm{PR}+\mathrm{RP}}<x_{\mathrm{PP}+\mathrm{RP}},\\ \mu >\mu_{13} & \Leftrightarrow & x_{\mathrm{PB}+\mathrm{RR}}<x_{\mathrm{PB}+\mathrm{RP}}\Leftrightarrow x_{\mathrm{PR}+\mathrm{RB}}<x_{\mathrm{PP}+\mathrm{RB}}, \end{array}$$

where $\mu_{0}=1+\frac{2}{\delta },\mu_{10}=1+\frac{4}{\delta (2+\delta )},\mu_{11}=1+\frac{4}{\delta (4+\delta )},\mu_{12}=1+\frac{2}{\delta (2+\delta )},\mu_{13}=1+\frac{4}{\delta (4+3\delta )}$, and *μ*_0_ > *μ*_10_ > *μ*_11_ > *μ*_12_ > *μ*_13_.

### A model of C-FO-linkage

The MIG assumes FO-SO-linkages. In this subsection, we analyze the replicator dynamics of a model that assumes C-FO-linkages instead of FO-SO-linkages. Accordingly, cooperators automatically provide first-order incentives, and thus, there is no CN, and no second-order incentive is needed in the MIG. When *y* is set to zero and all the parameters for the second-order incentives are also zero (*F*_*P*_ = *P*_*P*_ = *A*_*P*_ = *R*_*P*_ = *F*_*R*_ = *P*_*R*_ = *A*_*R*_ = *R*_*R*_ = 0), this new version, or an incentive game (IG), can be regarded as a model of the C-FO-linkages. We denote the three possible IG configurations as the P-type, R-type, and P+R type. The replicator equation of IG is

$$\begin{array}{c} \dot{x}=x\left(1-x\right)\left[\left(A_{1}+F_{1}-R_{1}+P_{1}\right)x-\left(c+P_{1}\right)\right]. \end{array}$$

We can devise a simple incentive game (S-IG) by using *μ*, *δ*, and *c* = 1. All S-IG types have two patterns of behavior depending on the values of (*μ*, *δ*). In one, there is a globally stable point (*x*, *z*) = (0, 1). In the other, another locally asymptotically stable point (*x*, *z*) = (1, 0) can exist. The existence condition for the basin of attraction at the stable point (1, 0) is $\mu >\frac{1}{\delta }$ for a P-type, $\mu >1+\frac{1}{\delta }$ for an R-type, or $\mu >\frac{1+\delta }{2\delta }$ for a P+R-type S-IG.

### Nonexistence of internal equilibrium

In this subsection, we prove that there is no equilibrium at any internal point on the 2-dimensional simplex Δ = {(*x*, *y*, *z*) : *x*, *y*, *z* ≥ 0, *x*+*y*+*z* = 1}. Assume that (x*, y*, z*) is an internal equilibrium. On that point, Eq (4) should be 0. This is because x* (y*, z*) is not zero, and thus, the function $\dot{x}$ ($\dot{y},\dot{z}$) can be divided by x*(y*, z*). Using $\frac{{\dot{y}|}_{y=y^{*}}}{y^{*}}=\frac{{\dot{z}|}_{z=z^{*}}}{z^{*}}=0$, one gets

$$\begin{array}{c} x^{*}=\frac{1}{A_{1}+F_{1}}. \end{array}$$

Similarly, using $\frac{{\dot{x}|}_{x=x^{*}}}{x^{*}}=\frac{{\dot{y}|}_{y=y^{*}}}{y^{*}}=0$, one gets

$$\begin{array}{ccc} & & z^{*}\left(R_{1}+P_{R}-P_{1}-P_{P}+\frac{R_{R}-P_{R}-R_{P}+P_{P}+A_{P}+F_{P}-A_{R}-F_{R}}{A_{1}+F_{1}}\right)\\ & & \;=R_{1}+P_{R}+\frac{R_{R}-P_{R}-A_{R}-F_{R}}{A_{1}+F_{1}} \end{array}$$ (6)

Table 3 shows the equations and solutions of z* in Eq (6) for all 24 types of S-MIG. The solutions of z* in the PP and RP types are not unique when *μδ* = 1. At that time, however, x* must be 1. Therefore, there is no equilibrium for both types when 0 < *δ* < 1.

**Table 3:** Equations and solutions of z* in Eq (6) for each type.

Type	Equation of z* in MIG	Solution of z* in S-MIG
P	−*P*_1_ z* = 0	0
R	*R*_1_ z* = *R*_1_	1
P+R	(*R*_1_−*P*_1_)z* = *R*_1_	no solution
PP	$z^{*}(-P_{1}-P_{P}+\frac{P_{P}+F_{P}}{F_{1}})=0$	not unique when *μδ* = 1
PR	$z^{*}(-P_{1}+\frac{-R_{P}+A_{P}}{F_{1}})=0$	0
PB	$z^{*}(-P_{1}-P_{P}+\frac{-R_{P}+P_{P}+A_{P}+F_{P}}{F_{1}})=0$	not unique when *δ* = 1
RP	$z^{*}(R_{1}+P_{R}+\frac{-P_{R}-F_{R}}{A_{1}})=R_{1}+P_{R}+\frac{-P_{R}-F_{R}}{A_{1}}$	not unique when *μδ* = 1
RR	$z^{*}(R_{1}+\frac{R_{R}-A_{R}}{A_{1}})=R_{1}+\frac{R_{R}-A_{R}}{A_{1}}$	1
RB	$z^{*}(R_{1}+P_{R}+\frac{R_{R}-P_{R}-A_{R}-F_{R}}{A_{1}})=R_{1}+P_{R}+\frac{R_{R}-P_{R}-A_{R}-F_{R}}{A_{1}}$	not unique when *δ* = 1
P+RP	$z^{*}(R_{1}+P_{R}-P_{1}+\frac{-P_{R}-F_{R}}{A_{1}+F_{1}})=R_{1}+P_{R}+\frac{-P_{R}-F_{R}}{A_{1}+F_{1}}$	no solution
P+RR	$z^{*}(R_{1}-P_{1}+\frac{R_{R}-A_{R}}{A_{1}+F_{1}})=R_{1}+\frac{R_{R}-A_{R}}{A_{1}+F_{1}}$	no solution
P+RB	$z^{*}(R_{1}+P_{R}-P_{1}+\frac{R_{R}-P_{R}-A_{R}-F_{R}}{A_{1}+F_{1}})=R_{1}+P_{R}+\frac{R_{R}-P_{R}-A_{R}-F_{R}}{A_{1}+F_{1}}$	no solution
PP+R	$z^{*}(R_{1}-P_{1}-P_{P}+\frac{P_{P}+F_{P}}{A_{1}+F_{1}})=R_{1}$	$\frac{2\mu }{1+\mu -2\mu \delta }(>1\;\text{or}\;<0)$
PR+R	$z^{*}(R_{1}-P_{1}+\frac{-R_{P}+A_{P}}{A_{1}+F_{1}})=R_{1}$	$\frac{2\mu }{\mu -1}(>1)$
PB+R	$z^{*}(R_{1}-P_{1}-P_{P}+\frac{-R_{P}+P_{P}+A_{P}+F_{P}}{A_{1}+F_{1}})=R_{1}$	$\frac{1}{1-\delta }(>1)$
PP+RP	$z^{*}(R_{1}+P_{R}-P_{1}-P_{P}+\frac{-P_{R}+P_{P}+F_{P}-F_{R}}{A_{1}+F_{1}})=R_{1}+P_{R}+\frac{-P_{R}-F_{R}}{A_{1}+F_{1}}$	no solution
PP+RR	$z^{*}(R_{1}-P_{1}-P_{P}+\frac{R_{R}+P_{P}+F_{P}-A_{R}}{A_{1}+F_{1}})=R_{1}+\frac{R_{R}-A_{R}}{A_{1}+F_{1}}$	$\frac{1+\mu }{2(1-\mu \delta )}(>1)$
PP+RB	$z^{*}(R_{1}+P_{R}-P_{1}-P_{P}+\frac{R_{R}-P_{R}+P_{P}+F_{P}-A_{R}-F_{R}}{A_{1}+F_{1}})=R_{1}+P_{R}+\frac{R_{R}-P_{R}-A_{R}-F_{R}}{A_{1}+F_{1}}$	$\frac{2\mu \delta }{1-\mu }(>1)$
PR+RP	$z^{*}(R_{1}+P_{R}-P_{1}+\frac{-P_{R}-R_{P}+A_{P}-F_{R}}{A_{1}+F_{1}})=R_{1}+P_{R}+\frac{-P_{R}-F_{R}}{A_{1}+F_{1}}$	$\frac{2\mu \delta +\mu -1}{2(\mu \delta -1)}(>1)$
PR+RR	$z^{*}(R_{1}-P_{1}+\frac{R_{R}-R_{P}+A_{P}-A_{R}}{A_{1}+F_{1}})=R_{1}+\frac{R_{R}-A_{R}}{A_{1}+F_{1}}$	no solution
PR+RB	$z^{*}(R_{1}+P_{R}-P_{1}+\frac{R_{R}-P_{R}-R_{P}+A_{P}-A_{R}-F_{R}}{A_{1}+F_{1}})=R_{1}+P_{R}+\frac{R_{R}-P_{R}-A_{R}-F_{R}}{A_{1}+F_{1}}$	$\frac{2\mu \delta +\mu -1}{2\mu \delta -\mu -1}(>1)$
PB+RP	$z^{*}(R_{1}+P_{R}-P_{1}-P_{P}+\frac{-P_{R}-R_{P}+P_{P}+A_{P}+F_{P}-F_{R}}{A_{1}+F_{1}})=R_{1}+P_{R}+\frac{-P_{R}-F_{R}}{A_{1}+F_{1}}$	$\frac{2\mu \delta +\mu -1}{\mu -1}(>1)$
PB+RR	$z^{*}(R_{1}-P_{1}-P_{P}+\frac{R_{R}-R_{P}+P_{P}+A_{P}+F_{P}-A_{R}}{A_{1}+F_{1}})=R_{1}+\frac{R_{R}-A_{R}}{A_{1}+F_{1}}$	$\frac{\mu +1}{1+\mu -2\mu \delta }(>1\;\text{or}\;<0)$
PB+RB	$z^{*}(R_{1}+P_{R}-P_{1}-P_{P}+\frac{R_{R}-P_{R}-R_{P}+P_{P}+A_{P}+F_{P}-A_{R}-F_{R}}{A_{1}+F_{1}})=R_{1}+P_{R}+\frac{R_{R}-P_{R}-A_{R}-F_{R}}{A_{1}+F_{1}}$	no solution

If *δ* = 1 is permitted, the PB and RB types have internal equilibria at all points on $x=x^{*}=\frac{1}{\mu }$. However, they are not stable. We will prove this and deal with the PB type as a representative. When *δ* = 1, Eq (1) in the PB type is

$$\begin{array}{c} \dot{x}=xz\left(\mu x-1\right)\left(3-2x\right),\;\dot{y}=yz\left(\mu x-1\right)\left(1-2x\right). \end{array}$$

Now let us analyze the local stability of the point (*x*, *y*, *z*) around the equilibrium point (x*, y*, z*). Let *ε*_*x*_ = *x*−x*, *ε*_*y*_ = *y*−y* and *ɛ* = (*ε*_*x*_, *ε*_*y*_)^*T*^, where ^*T*^ means transposition. As a result of the linearization of the dynamics, $\frac{dɛ}{dt}=Mɛ$ where

$$\begin{array}{c} M=(\begin{array}{cc} \mu x^{*}z^{*}\left(3-2x^{*}\right) & 0\\ \mu y^{*}z^{*}\left(1-2x^{*}\right) & 0 \end{array}), \end{array}$$

because μx*−1 = 0. The eigenvalues of *M* are *λ* = 0, z*(3−2x*). These are non-negative, and thus, any internal equilibrium is non-isolated and unstable. The eigenvalues in the case of the RB type are *λ* = 0, z*+2(x*+y*)(y*+z*), and thus, the same conclusion is reached.

### Local stability of the line *z* = 0 in the P, PP, PR, and PB types

In this subsection, we analyze the local stabilities of the line *z* = 0 in the P, PP, PR, and PB types of S-MIG. Here, we deal with the P-type S-MIG as a representative. Eq (1) becomes

$$\begin{array}{c} \dot{x}=xz\left[\mu \delta x-\delta \left(y+z\right)-1\right],\;\dot{y}=yz\left[\mu \delta x+\delta x-1\right]. \end{array}$$

As is shown in the previous subsection, the linearization of the dynamics around the equilibrium point (*x*, *y*, *z*) = (x*, y*, 0) leads to $\frac{dɛ}{dt}=Mɛ$, where *ε*_*x*_ = *x*−x*, *ε*_*y*_ = *y*−y*, *ɛ* = (*ε*_*x*_, *ε*_*y*_)^*T*^, and

$$\begin{array}{c} M=(\begin{array}{cc} x^{*}\left[1+\delta -\left(\mu +1\right)\delta x^{*}\right] & x^{*}\left[1+\delta -\left(\mu +1\right)\delta x^{*}\right]\\ y^{*}\left[1-\left(\mu +1\right)\delta x^{*}\right] & y^{*}\left[1-\left(\mu +1\right)\delta x^{*}\right] \end{array}). \end{array}$$

The eigenvalues of *M* are *λ* = 0, 1−μδx*, and thus, the whole line *z* = 0 consists of fixed points and it is stable (unstable) when $x>\frac{1}{\mu \delta }$ ($x<\frac{1}{\mu \delta }$) as well as the PP, PR, and PB types.
